# Research Progress of Chitosan-Based Biomimetic Materials

**DOI:** 10.3390/md19070372

**Published:** 2021-06-27

**Authors:** Zhaoyu Zhang, Lingyu Zhang, Chengpeng Li, Xiangyu Xie, Guangfa Li, Zhang Hu, Sidong Li

**Affiliations:** Faculty of Chemistry and Environmental Science, Guangdong Ocean University, Zhan Jiang 524088, China; zhangzy227@163.com (Z.Z.); zhangly175@163.com (L.Z.); lcp0802@126.com (C.L.); xiexiangyu1@stu.gdou.edu.cn (X.X.); lgf0719@163.com (G.L.); sidongligdou@163.com (S.L.)

**Keywords:** chitosan, biomimetic materials, mussels, cell matrix

## Abstract

Chitosan is a linear polysaccharide produced by deacetylation of natural biopolymer chitin. Owing to its good biocompatibility and biodegradability, non-toxicity, and easy processing, it has been widely used in many fields. After billions of years of survival of the fittest, many organisms have already evolved a nearly perfect structure. This paper reviews the research status of biomimetic functional materials that use chitosan as a matrix material to mimic the biological characteristics of bivalves, biological cell matrices, desert beetles, and honeycomb structure of bees. In addition, the application of biomimetic materials in wound healing, hemostasis, drug delivery, and smart materials is briefly overviewed according to their characteristics of adhesion, hemostasis, release, and adsorption. It also discusses prospects for their application and provides a reference for further research and development.

## 1. Introduction

Chitin (CT), the second most abundant natural polysaccharide on earth, after cellulose, is a major structural component in the exoskeletons of a variety of organisms, including protists, diatoms, sponges, arthropods, molluscs, insects, and arachnids, especially seafood, such as shrimp and crab [1,2,3,4,5]. Traditionally, CT was isolated on a large scale from the exoskeletons of fungal organisms and crustaceans. Its extraction includes the chemical method, microbial fermentation, and bioenzymatic hydrolysis [1,6,7]. Chitosan (CS), a deacetylated derivative of chitin, has net cationicity and a variety of functional groups, which can form electrostatic complexes or multilayer structures with other negatively charged substances or natural polymers. In recent years, it has been widely used in many fields, such as tissue engineering, wound healing, drug transportation, adhesives, and adsorption materials, due to its good biocompatibility and biodegradability, safety and non-toxicity, broad-spectrum antibacterial properties, and hemostasis. Additionally, it is easy to process it into gels, membranes, nanofibers, stents, and other forms [8,9,10,11,12,13,14].

**Figure 1:**
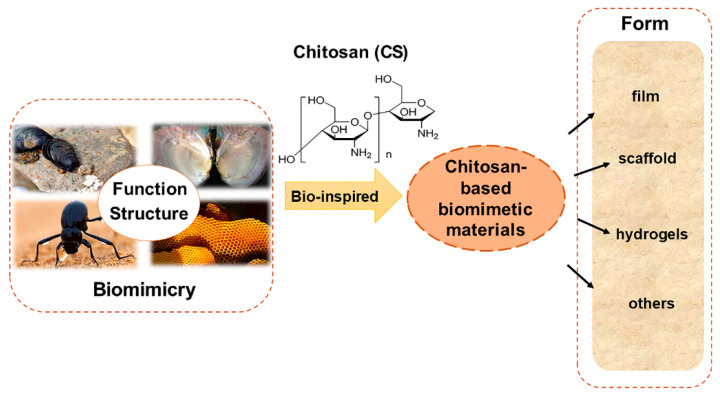
The chitosan-based biomimetic materials and the forms of material prepared.

Bionics is an emerging discipline that studies the structure, function, and optimization of biomaterial systems through the intersection of biology, chemistry, and physics. It uses active substances derived from nature to design various structural and functional materials through the principle of bionics. In recent years, it has become a rapidly developing research field [15,16,17]. In most cases, bionics does not mean directly copying the structure of biological materials; instead, guiding principles are extracted from biological systems for the artificial synthesis of functional materials with relevant characteristics [18,19,20]. This review focuses on the imitation of unique structures and functions of some natural organisms, such as bivalves, biological cell substrates, desert beetles, and honeycomb structures of bees, and the preparation of functional materials with related properties and their derivatives, using CS as the matrix material (Figure 1). The applications of biomimetic materials in wound healing, hemostasis, drug delivery, and smart materials were reviewed according to their unique characteristics of adhesion, hemostasis, release, and adsorption. Meanwhile, to better understand the role of CS in biomedicine, tissue engineering, adsorption materials, and other fields, the prepared CS-based biomimetic materials were briefly summarized according to their forms, including scaffold, hydrogel, film, and composite material (Table 1).

## 2. Biomaterials Imitating Bivalves

### 2.1. Imitation Mussel Adhesive Material

Marine mussel organisms possess a solid structure to enable strong, persistent adhesions to various materials in humid environments. It is mediated by a mussel-foot-protein (MFPS) sequence structure containing a large amount of 3,4-dihydroxy phenyl-l-alanine (DOPA) [73,74,75]. The catechol group is critical to its adhesion mechanism and is considered an ideal candidate for constructing solid matrix adhesion materials [46]. The corresponding CS-catechol conjugate (CS-C) has antifluid adhesion properties and hemostatic ability [76,77]. It can be used as bio-printing materials [78], drug delivery libraries [79], and for nanoparticle surface functionalization [80]. 

#### 2.1.1. Hemostatic and Tissue Adhesive Material

**Figure 2:**
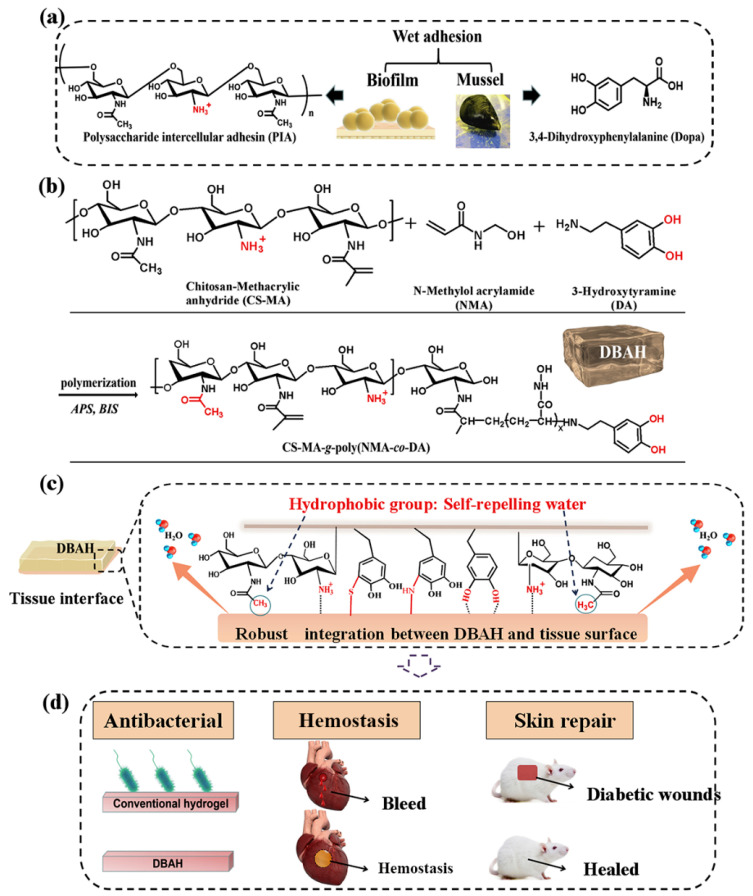
Schematic illustration of design strategy of an engineered biofilm and mussel-inspired dual-bionic adhesive hydrogels (DBAH) and its application for sealing hemostasis and wound healing. (**a**) The structure of polysaccharide intercellular adhesin (PIA), derived from biofilm, and DOPA, derived from mussel protein, which play a key role in wet adhesion; (**b**) A biometic biopolymer chitosan, grafted with methacrylate (CS-MA) from PIA; and dopamine, a catecholamine containing a catechol group of DOPA, was conjugated with NMA for hydrogel formation; (**c**) Schematic illustration of strong underwater bioinspired adhesion base on the self-repelling water function of CS-MA. (**d**) The multifunctional properties and potential application in in vivo hemorrhage and diabetic wound healing with antibacterial performance. Reproduced with permission from [27], Copyrighter Elsevier 2020.

CS-C, inspired by the mussel-adhesive protein, is the closest mimic of mussel adhesion proteins. It was obtained by grafting catechol derivatives to the amino groups of the CS backbone. In addition, CS-C has been widely exploited as general hemostatic materials, adhesives, and nano-particle composites due to its inherent property of immediate complexation with serum proteins. Shin et al. developed self-sealing hemostatic needles and adhesive coatings. The surface of the needle was coated with partially crosslinked catechol-functionalized CS, which could immediately prevent partial bleeding of the injection and may therefore help to prevent complications associated with bleeding in more clinical settings [26]. Combined with the characteristics of biofilms, Han et al. developed a novel dual bionic adhesive hydrogel (DBAH) by free radical polymerization. Specifically, methacrylate (CS-MA), dopamine (DA), and N-methylol acrylamide (NMA) were grafted on CS molecules (Figure 2). Compared with the conventional commercial medical adjuvants, these hydrogels presented excellent hemostatic capability under wet and dynamic motion in vivo [27]. Zhang et al. [28] prepared a hydroxybutyl chitosan-CS-dopamine composite hydrogel with the dopamine self-polymerization method, which was used to dress in vitro hemostatic wounds. The results showed that the composite hydrogel had temperature sensitivity, low hemolysis rate, and a short blood clotting time. Also inspired by MFPS, Han et al. synthesized a series of CS-grafted polypeptide copolymers that showed good biodegradability, low cytotoxicity, and good hemostatic properties, which could also promote the healing of skin wounds and fractures [29]. Kim et al. [30] investigated the enhancement of mucoadhesion properties of CS by catechol coupling. The retention ability of CS-C in the gastrointestinal tract was improved by the formation of irreversible catechol-mediated crosslinking with mucin. Inspired by the current obstacles in oral cavity drug delivery, Ryu et al. developed porous spongy-like adhesive materials, a freeze-dried form from the CS-C solution called “Chitoral”. Chitoral instantly dissolved upon contacting with saliva in the oral cavity and then formed intermolecular complexes with oral mucins, which were rapidly transformed into an adhesive hydrogel-like material through the synergistic action of covalent cross-linking and physical entanglement [31]. Based on the previous research [81,82], Zeng et al. [32] integrated various functions of other materials to design an injectable double cross-linked hydrogel adhesive, based on tetra-succinimidyl carbonate polyethylene glycol (PEG-4S), and thiol-grafted mussel inspired catechol conjugated CS (CSDS). Related performance evaluation results showed that the mechanical and adhesion properties of double-crosslinked hydrogel have been significantly improved. He et al. fabricated an injectable two-component hydrogel prepared from catechol and methacrylate modified CS/gelatin to solve the current challenges faced by medical adhesives [33]. Instantaneous gelation is required for the practical applications of CS-C as tissue adhesives. Ryu et al. [34] prepared a temperature-sensitive injectable hydrogel with high adhesion by mixing CS-C with thiolated Pluronic F-127. The addition of Pluronic F-127 effectively shortened the gelation time and improved the adhesion property of CS-based hydrogel, which was expected to be used in tissue engineering adhesives and antibleeding materials. 

**Figure 3:**
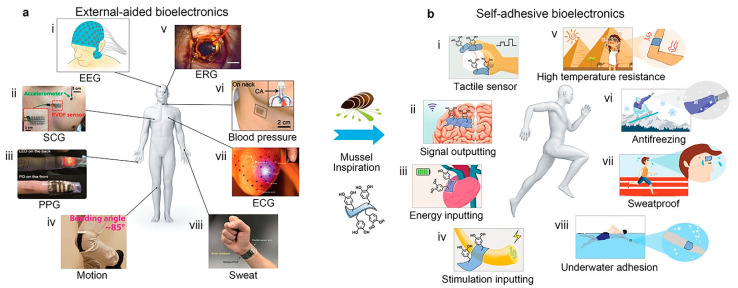
The mussel-like strategy provides novel approaches for wearable/implantable bioelectronics to shift from external auxiliary fixation to convenient and reliable self-adhesion. Reproduced with permission from [84], Copyrighter Wiley 2017.

Despite its versatility, the practical application of CS-C in clinical practice is limited because it is a highly positively charged polymer that can cause increased severe protein adsorption and trigger an immune response in the body [26,83]. To ameliorate the adverse immune response between polymer materials and proteins, Park et al. proposed a catechol-conjugated glycol CS hemostatic hydrogel inspired from mussel adhesive proteins and compared them with non-glycol CS-C hydrogels to evaluate their immune response, cytotoxicity, adhesion properties, and hemostatic ability. The results showed that glycol CS-C significantly attenuated the immune response. However, the tissue adhesion and hemostatic ability of glycol CS-C were not dramatically improved, and it was speculated that the finding was likely due to the antibiofouling effect of the ethylene glycol group and the reduction of immune cell adhesion [35]. Although significant progress has been made in bioelectronics research in recent years, it is still challenging for self-adhesive bioelectronics to adhere to human tissues and achieve signal detection without external aids. So far, there are only a few reports on self-adhesive bioelectronics, and they are primarily limited to collecting signals on the skin. Applications such as tactile sensors and implanted neural interface electrodes have not been reported. Introducing self-adhesion into currently available bioelectronic materials remains difficult due to the complex application environment and strict requirements, including biocompatibility, biological stability, and wet adhesion ability in the body fluid environment. Xie et al. [84] proposed an approach to regulate the dynamic equilibrium mechanism of phenolquinone redox based on a mussel-like strategy and developed a series of new biomedical hydrogels endowed with bioelectronic self-adhesion and other functional properties, such as super mechanical, self-repair, transparency, antibacterial, high-temperature resistance, frost resistance, and underwater adhesion (Figure 3). Kim et al. [36] provided a new strategy to design marine biomaterial hydrogels synthesized from catechol, CS, and diatom. It was highly stretchable and self-healing and can be utilized for various applications, including stretchable power sources, wearable electronics, and health monitoring systems with artificial intelligence. 

#### 2.1.2. Drug Delivery and Active Compound Release Material

**Figure 4:**
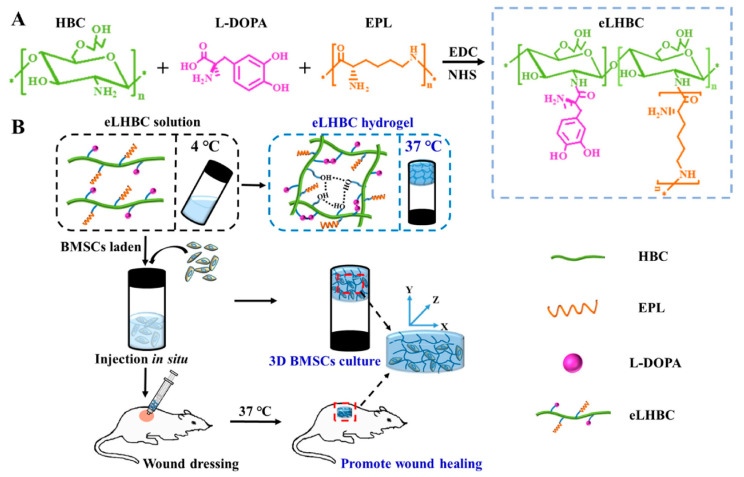
(**A**) Synthesis scheme of eLHBC conjugate via the binding of carboxyl groups ofl-DOPA and EPL to the NH_2_ groups of HBC. (**B**) The formation of eLHBC at 37 °C. BMSCs encapsulated into eLHBC solution at 4 °C and eLHBC as 3D BMSCs culture matrix at 37 °C (BMSCs ⊂ eLHBC). The BMSCs ⊂ eLHBC injected with a syringe on the dorsal wound site of rats and used as wound dressing. Reproduced with permission from [39], Copyrighter Elsevier 2021.

The application of hydrogels in clinical wound treatment-related fields has been reported frequently, but the exploration of intelligent hydrogels that can respond quickly and accurately to complex and multiple stimuli is still a promising strategy. Hu et al. [37] reported a novel design strategy for a double-crosslinked smart hydrogel for local release treatment of bacteria-infected diabetic wounds. First of all, they completed the first cross-linking to prepare hydrogel by a Schiff base reaction between the amino groups (-NH_2_) in the CS quaternary ammonium salt (HTCC) and the aldehyde groups (-CHO) in the oxidized dextran-dopamine (OD-DA). The second step of cross-linking was completed by the formation mechanism of catechol-catechol adducts in hydrogels [85]. Finally, based on their design strategy, they used a simple hybrid approach to impart antimicrobial and angiogenic efficacy to the hydrogels by loading AgNPs with the antiangiogenic drug deferriamine (DFO). To combat bacterial infections and achieve safe and controllable wound healing, Gao et al. mixed glycol CS (GC) with polydopamine (PDA) nanoparticles (NPs) loaded with ciprofloxacin (Cip) to produce a PDANP-Cip/GC injectable hydrogel. Under near-infrared light irradiation, this gel cooperated with Gel-Cip to accelerate Cip release and activated photothermal PDA NPs to achieve an efficient sterilization effect and promoted wound healing. Experimental analysis has confirmed its biological safety in vivo [38]. In view of the shortcomings of previously prepared thermosensitive hydroxybutyl CS (HBC) hydrogel carrying antibiotics and growth factors, Tian et al. [39] introducedl-DOPA to improve the adhesion and mechanical properties of HBC on wet tissue surfaces. Additionally, ε-Poly-l-lysine (EPL), an antimicrobial peptide, was introduced to improve its antimicrobial ability in a neutral environment and reduce the potential harm caused by the introduction of antibiotics. By taking advantage of EPL, (l-DOPA)-(EPL)-HBC hydrogels (eLHBC) displayed highly efficient antimicrobial activity against *Escherichia* *coli* and *Staphylococcus* *aureus*. The bone marrow mesenchymal stem cells (BMSCs) encapsulated into eLHBC could secrete growth factors and promote the migration of fibroblasts. The potential biomaterials of BMSCs ⊂ eLHBC could promote wound healing and skin tissue regeneration (Figure 4). Zhang et al. [40] prepared a CS-based thermo-sensitive hydrogel loading oyster peptide (CS-C/OP/β-GP) with a porous three-dimensional network and rapid hemostasis effect by combining catechol-functionalized CS and oyster peptide (OP). The safety evaluation confirmed that the CS-C/OP/β-GP hydrogel was non-cytotoxic to L929 fibroblasts. In vitro experiments showed that CS-C/OP/β-GP hydrogel could absorb a large amount of water from plasma to concentrate the blood due to its porous structure, polycationic characteristics, and good water absorption capacity, thereby achieving rapid hemostasis. It is speculated that CS-C/OP/β-GP hydrogel had good application prospects in the field of medical hemostasis.

#### 2.1.3. Functional Composite Material

**Figure 5:**
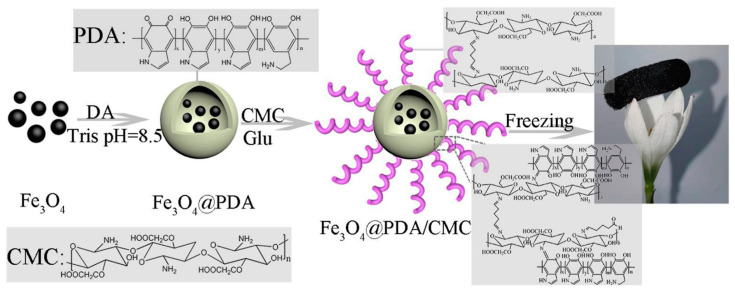
Schematic illustration of the preparation of Fe_3_O_4_@PDA/CMC aerogel (polydopamine (PDA), dopamine (DA), Tris (hydroxymethyl)aminomethane (Tris), Carboxymethyl chitosan(CMC), and Glutaraldehyde (Glu)). Reproduced with permission from [64], Copyrighter Elsevier 2021.

The properties of mussels and CS molecules can be exploited to obtain composite materials with tunable properties. To explore the potential of catechol-functionalized CS, Ghadban et al. [41] used metal-catechol coordination to design pH-sensitive and magnetic-responsive hydrogels and then incorporated iron oxide (γ-Fe_2_O_3_) MNPs into the formulation to expand its functions. This strategy made the gel magnetically responsive, increased the mechanical response, and enabled the control of drug release kinetics. Ni et al. prepared a new type of catechol-CS (CCS)-iron oxide NP (IONP) composite material that had a firm surface affinity and significantly improved immobilization under optimal conditions. The loading capacity and remaining activity of the enzyme provided an improved platform for bio-macromolecule immobilization [61]. Zeng et al. [62] combined mussel excitation chemistry and the Michael addition reaction to modify the surface of carboxymethyl CS on multiwalled carbon nanotubes (CNTs) to produce CNT-PDA-CS. Dopamine (DPA) contains a large number of active groups, such as catechol, amino, and (im)amino groups. Under mild alkaline conditions, polydopamine films can be formed on various inorganic and organic materials through spontaneous oxidation polymerization [86,87]. Mussel-inspired polydopamine (PDA)-related materials have attracted interest in the making of multifunctional materials. Wang et al. [63] fabricated a novel magnetic hybrid nano-biosorbent (Fe_3_O_4_@PDA@CS) via Schiff base reaction, which had a strong adsorption capacity and efficient removal of dyes and metals. Lei et al. [64] prepared Fe_3_O_4_@PDA/CMC aerogel by the Schiff base reaction between PDA and CMC using a simple method of introducing the polydopamine Fe_3_O_4_NPS surface using a mussel-inspired chemistry coating strategy. The experimental results showed that Fe_3_O_4_@PDA/CMC aerogel adsorbent had excellent magnetic properties and high adsorption capacity (Figure 5). Therefore, it was a high efficiency, economical price, and environmental protection material expected to remove dyes from an aqueous solution. Hydroxyapatite (HAP, Ca_10_(PO_4_)_6_(OH)_2_) has been successfully used in many biomedical fields due to its unique biological activity. The bioactive properties of HAP and the enhancement of the mechanical strength of CS provide a new approach for the treatment of damaged hard tissue. Szatkowski et al. [65] selected calcium chloride (CaCl_2_) and disodium hydrogen phosphate (Na_2_HPO_4_), which are rarely described as precursors of mineral phases in CS composites in literature, as sources of calcium and phosphorus, or CS as the organic phase to prepare HAP/CS composites. By comparing the HAP/CS (mineral and organic) biomaterials prepared in different proportions, they found that the HAP/CS ratio of 85/15 had the best morphological characteristics (high specific surface area and porosity). In addition, energy dispersive spectrometer (EDS) analysis showed that the precipitated HAP had a calcium/phosphorus ratio similar to that of natural minerals, which confirmed the feasibility of its synthesis process. Li et al. [21] proposed a feasible and effective covalent method for immobilization of CS onto the surface of porous poly(ε-caprolactone) (PCL)/bioactive glass (BG) composite scaffolds using a mussel-inspired PDA coating. The experimental results showed that the scaffolds had obvious advantages compared with the simple physical adsorption CS scaffold, with excellent potential in orthopedic repair. 

### 2.2. Imitation Pearl Layer Structure Material

Biomineralization refers to a biological process that forms highly ordered biological materials, such as shells, pearls, enamel, or bones, through the interaction of specific surface binding at the organic-inorganic interface. Because of their specific combination, biominerals have a hierarchical layered structure, as well as mechanical and physical properties, which are more suitable for diversified functional purposes. Nacre is a type of mineralized tissue deposited by many mollusk species (Bivalves and cephalopods) to build the inner layers of their shells. Mature nacre consists of thin layers (~30 nm) of matrix and thicker layers (~500 nm) of the calcium carbonate mineral aragonite (lamellae). The matrix layer accounts for about 5%, and calcium carbonate accounts for about 95%. Although proteins account for a small part of the content of biominerals, they directly participate in controlling the growth of biological crystals, thereby enhancing the mechanical properties of biominerals [47,88,89,90,91,92]. In recent years, researchers have used a variety of innovative techniques to simulate the nacreous layer microstructure and produce materials with good mechanical properties. 

#### 2.2.1. Composite Film Material

**Figure 6:**
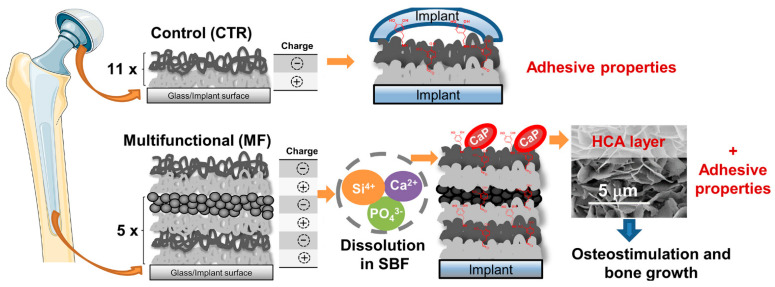
Schematic illustration of the different multifunctional (MF) and control (CTR) LbL coatings (11×: the number of repetitions required for the CTR group to alternately immerse the substrate in the oppositely-charged polyelectrolyte solutions to produce LbL coatings with 11 bilayers, i.e., 22 layers; 5×: the number of repetitions required for the MF group to alternately immerse the substrate in the oppositely-charged polyelectrolyte solutions to produce LbL coatings with 22 layers). Reproduced with permission from [50], Copyrighter Elsevier 2020.

Through the bottom-up continuous deposition of organic and inorganic layers under ambient environmental conditions, Bonderer et al. [93] obtained layered hybrid films combining high tensile strength and ductile behavior. Ma et al. successfully deposited CS and PDA on silk fibroin nanofibers through layer-by-layer self-assembly (LbL) technology to modify them to have antibacterial ability. Surface morphology and composition analyses of the cell-compatible LbL structural film confirmed successful deposition. The wet tensile modulus of the film increased from 2.16 MPa (pure silk fibroin film) to 4.89 Mpa [48]. Using freeze induced assembly and hot-pressing methods, Chen et al. [49] toughened and modified delignificated nano-cellulose (DNLC) by the synergistic effect of CS and MoS_2_ and prepared the high-performance ternary lignocellulose nacres. Additionally, Almeida et al. [50] proposed multifunctional (MF) CS/hyaluronic acid (HA) LbL films developed by the dip-coating technology. They alternately combined the inorganic nanoparticles and bioactive glass nanoparticles (BGNP) with catechol-functionalized biopolymeric layers CS and HA to obtain MF films, which could be used in bone tissue engineering because of their ability to create an environment compatible with osteogenesis (Figure 6). Abba et al. [51] used CS as the matrix material (mortar) and alumina sheets as the reinforcing particles (brick) to prepare the nacreous microstructured mixed film material. The effects of inorganic to organic matter ratios and relative humidity on mechanical properties were studied. The results showed that the relative humidity of the environment had a significant effect on the measured mechanical properties. Yao et al. [46] prepared brick-and-mortar CS-layered dihydroxide hybrid films by sequential dipping coating and LbL technology, which had high mechanical properties and tensile strength of 160 MPa (higher than that of natural brick-and-mortar films). Subsequently, they developed a novel approach for manufacturing CS-montmorillonite (MTM) biomimetic composite membranes with a self-assembly method caused by vacuum filtration or water evaporation. The hybridized CS-MTM building blocks were arranged into a pearl layered structure composite material. The film had high performance in terms of mechanical properties, light transmittance, and fire resistance [52].

#### 2.2.2. Nanocoatings and Other Composite Material

**Figure 7:**
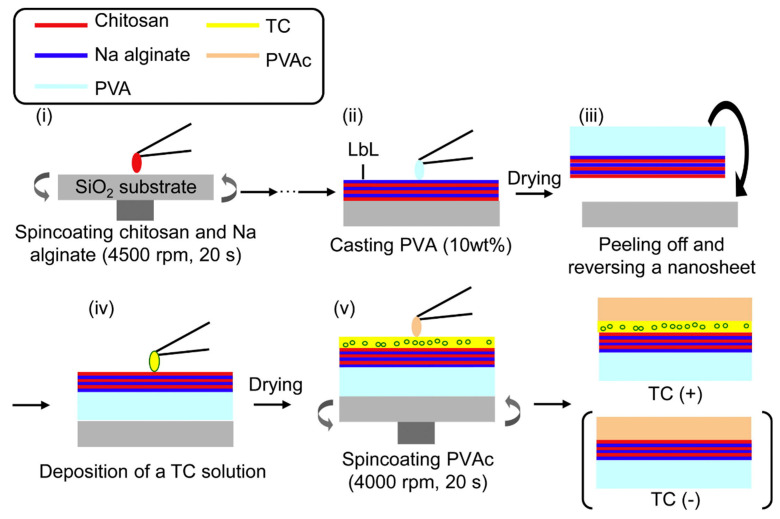
Preparation scheme for TC-loaded nanosheets. (**i**) Chitosan and sodium alginate were alternately spin coated. (**ii**) PVA was cast onto the LbL nanosheet. (**iii**) After drying PVA as a supporting layer, the nanosheet was peeled off with PVA and reversed onto the substrate. (**iv**) TC solution deposited on nanosheet. (**v**) After drying to make a TC layer, PVAc was spin coated onto the new layer. Reproduced with permission from [55], Copyrighter Elsevier 2012.

Using carboxymethyl CS (CCS) and MMT, Xie et al. [53] fabricated super-efficiency fire-safe nanocoating via one-step self-assembly, which showed well-arranged nacre-like microstructure and high transparency. Fang et al. demonstrated a convenient approach to fabricate a nacre-mimetic flame-retardant system by the LbL method using MMT as matrix and CS as mortar to simulate the structure of the nacreous layer. The prepared paper had excellent flame retardant properties [54]. It is noteworthy that visualization of the injured tissue may allow prompt and appropriate wound care in burn-injured patients. Based on the previous reports [94], Saito et al. [55] loaded antibiotics (tetracycline, TC) on nanosheets and developed TC nanosheets with high transparency and fluorescence that could help monitor burn care management (Figure 7). The prepared TC-nanosheet was composed of three layers (LbL/TC/PVAc): LbL made of CS and sodium alginate (SA) as a bottom layer, TC as an antibiotic layer, and poly(vinyl acetate) as a hydrophobic barrier layer.

The versatile technique used to construct the antibiotic-loaded nanosheets is applicable to other drugs with appropriate solubility with that of membrane components. Based on the principle of bio-adhesion/anti-adhesion, Yuan et al. [56] developed an organ-like biological coating chip with self-repair and antioxidant functions through LbL self-assembly, which was used for cell sorting, capture, and on-demand release. In this work, two coatings with opposite functions on enhancing cell adhesion coatings were fabricated. Adherent-enhancing coatings (CROD coatings) were prepared from oxidized alginate grafted with carboxymethyl CS modified with RGD polypeptides, and an anti-adhesion coating (PANM coatings) was prepared mainly from 2-methylacrylloxyethyl phosphorycholine (MPC). Metal ions were introduced into the artificial nacre to form new chemical bonds and reinforce the interface interaction. Chen et al. systematically studied the influence of different metal ions on the binary system. It was found that different metal ions with various sizes and charges would affect machine performances. Through the introduction of Mg^2+^, the strength and toughness of MTM-CS ternary artificial nacre could reach 200 MPa and 40 MJ m^−3^, respectively, and its toughness was 20 times that of natural nacre [57]. Combining the layered structure of brick-and-mortar and the biological mineralization process, Zhang et al. [66] constructed a hydroxyapatite/CS (HA/CH)-layered composite material to remove heavy metal Pb(II) from continuous flowing wastewater. The composite material contained a microstructure similar to a plate tower; large pores between layers facilitated the transfer of continuous flowing wastewater and the separation of adsorbent and water. Ruan et al. designed an organic matrix composed of CS and cisbutenediolic acid (maleic acid, MAc) to simulate the function of a nacreous matrix and then generated a layered montmorillonite matrix of composite materials. Hydroxyapatite with a multiscale hierarchical structure was synthesized using layered montmorillonite-CS composites as precursors via a topotactic phase transformation process [67].

## 3. Imitation Extracellular Matrix Material

The extracellular matrix (ECM) is important for guiding cellular development and maintaining the desired phenotype. It is both the structural basis of cells and the source of three-dimensional biochemical and biophysical signals that trigger and regulate cell behavior [95,96,97,98]. 

### 3.1. Functional Cell Micro-Environment Material 

**Figure 8:**
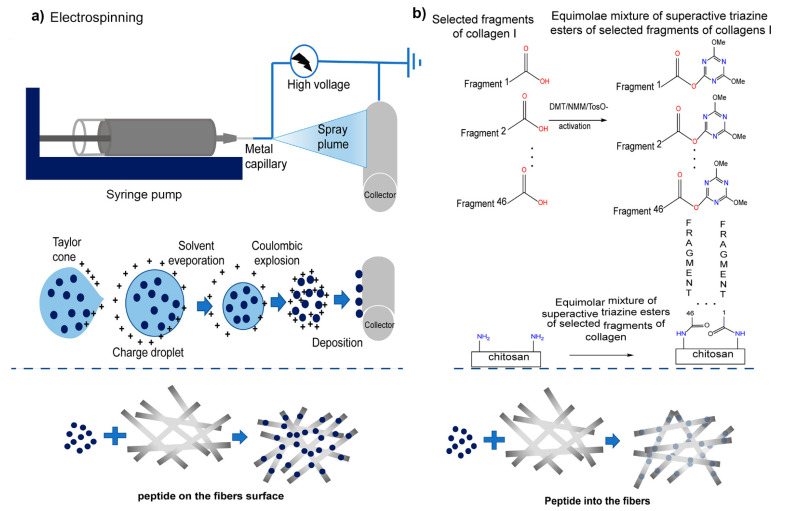
Scheme of applied modification methods of fibrous chitosan substrates: Physical (**a**) electrospinning was used to apply the solution of human collagen I fragments to the CS non-woven fabric through the potential difference between the nozzle and the collector. The resulting CS with physically embedded fragments of human collagen I (CS/F K1) were fixed using lyophilization. Chemical (**b**), the coupling reagent, and NMM were added into the peptides solution to activate the peptide, and the non-woven sheet of CS was immersed in the solution containing active peptides to complete the reaction. After washing and drying, the covalent link fragment of the non-woven sheet of CS and human collagen I (CS/C K1) was obtained. Reproduced with permission from [68], Copyrighter Elsevier 2020.

Materials that can imitate the shape and function of natural ECM in vivo are being developed for in vitro research in the field of cell micro-environment engineering [99,100,101,102]. Construction of functional tissues relies on the structural environment, cell–biomaterial interactions, and incorporated biological signals [103]. In this sense, hydrogels can easily adjust their physico-chemical (electrical charge and pore size) [104] and mechanical properties [105], which natively lead to cellular function. María et al. combined a hydrogel backbone network composed of CS and hyaluronic acid with a terpolymer containing catechol; this hybrid system had the advantage of the two polymers crosslinked with Fe to form an interpermeable polymer network (IPN). When used as a wound dressing, this IPN can constitute an ECM simulation platform with high cell affinity and bioactivity. Combined with the controlled release of catechin, it promoted the tissue regeneration process and contributed to wound healing [42]. Ewa et al. [68] modified the surface of CS fibers with fragments of human collagen I (10–15 amino acids) by physical (water absorption during electrospraying) and chemical (amide and peptide groups) activities to obtain a complex of glycosaminoglycans (GAG) and peptides similar to those present in the cell matrix (Figure 8). Subsequently, the effect of different modification methods on the fiber was evaluated. Compared with physical modification, the results showed that the chemical modification made the peptide evenly distributed on the fiber without changing its shape. To promote the development of organ-on-a-chip and other physiologically-relevant biomembrane fields, Rosella et al. [43] presented a microfluidic platform for the synthesis of biomembranes during gelation and studied its role as an extracellular matrix support. High-throughput studies on biomembranes were prepared with different biopolymer materials to characterize the relationship between the different conditions imposed on them, thereby revealing their biological application potential.

### 3.2. Bone Tissue Engineering Material

**Figure 9:**
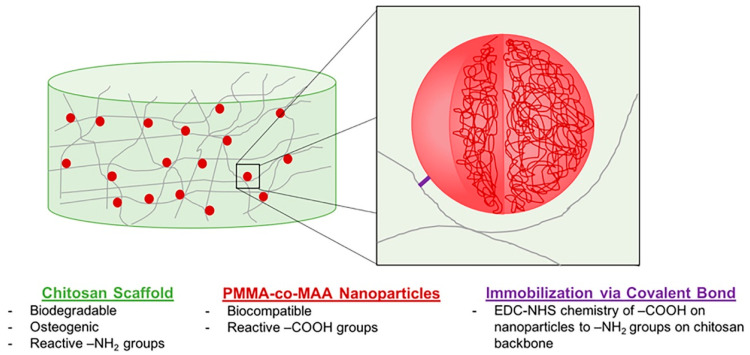
Design of a two-phase NP-scaffold system relying on the immobilization of P(MMA-co-MAA) NPs to a CS scaffold backbone for the sustained delivery of growth factors in bone tissue engineering applications. Reproduced with permission from [24], Copyrighter Wiley 2020.

Orthopedic biomaterials or coatings with ECM-like nano-features can induce ideal interactions between bone tissue and the implant surface. CS-gelatin (G) composite materials have promising bone tissue engineering possibilities because they combine the cellular adhesion of G with the antibacterial properties of CS [106,107,108]. Altuntas et al. [58] prepared nanocrystalline CS:G films with ordered nanopore arrays that were developed using anodic alumina molds via a drop-casting approach. Experiments demonstrated that these nanopillared films had good bactericidal properties and the ability to induce early osteogenic differentiation, making them a promising antibacterial coating material for planting. Tangprasert et al. prepared a gelatin/CS/compounded calcium phosphate (Gel:CS:CCP) hydrogel to simulate the extracellular matrix of calcified soft tissues and then designed an ex-vivo model for evaluating tissue formation. The results showed that the molecular structure and morphology of the self-organized hydrogel in Gel: CS: CCP (1:1:0.1) was similar to the extracellular matrix formed by in-situ bone. Its physical and biological properties enhanced cell viability and proliferation [44]. It was speculated that the remarkable characteristics of CS and the ability of graphene oxide (GO) to refine and repair major bone defects could induce and support bone tissue formation. To promote one novel scaffold based on a natural compound of CS and GO, Dinescu et al. [22] explored the potential of a new scaffold for tissue engineering applications and regenerative medicine. Drawing lessons from the necessary support structure for tendon regeneration, Sundaram et al. presented a CS hydrogel scaffold reinforced with a twisted poly(l-lactic acid) aligned microfibrous bundle to mimic tendon extracellular matrix [23]. De Witte et al. [24] fabricated a biodegradable, osteoconductive, porous chitosan scaffold via the freeze-drying method, synthesized poly(methyl methacrylate-co-methacrylic acid) nanoparticles, and immobilized to the scaffold via carbodiimide-crosslinker chemistry. Fluorescence imaging results showed that the nanoparticles were wholly retained in the scaffold for up to 4 weeks, had good biocompatibility, and did not negatively affect human umbilical vein endothelial cells (Figure 9). To find effective bone tissue regeneration materials based on mesenchymal stem cells, Zhao et al. [45] developed an injectable or bio-printed carboxymethyl chitosan (CMCh) and amorphous calcium phosphate (ACP) composite NP hydrogel (designated CMCh-ACP hydrogel). It was the first to demonstrate that pH changes can be used to control the assembly of polymer-stabilized ACP NPs to form elastic hydrogels. Their findings strongly suggested that CMCh-ACP hydrogels may be developed into novel scaffolds for stem-cell-based bone tissue engineering.

## 4. Imitation Desert Beetle Material

**Figure 10:**
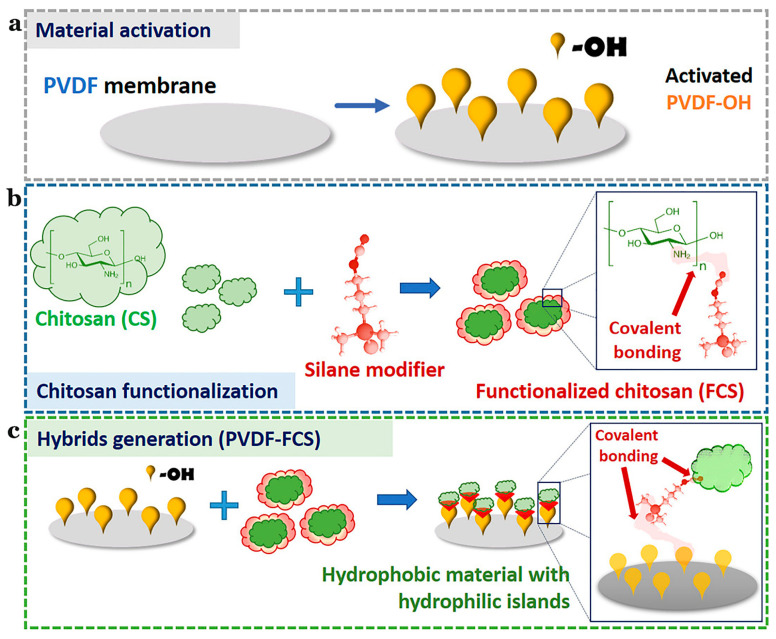
Schematic illustration of the preparation of PVDF-FCS. (**a**) The reaction for chitosan functionalization. (**b**) CS was anchored to pre-activated PVDF membranes by urea linker via salinization. (**c**) The generation of the hybrid materials. Reproduced with permission from [59], Copyrighter Elsevier 2020.

The Namib desert beetle can collect drinking water from fog-laden wind. This is because the insect’s bumpy surface alternates between waxy hydrophobic concave areas and non-waxy hydrophilic convex areas. The unique wettability of this pattern allows it to capture and coalescence tiny water droplets in the fog in the hydrophilic bulge area, then to the hydrophobic smooth area, and finally to the insect’s mouth [109,110,111,112,113]. Combining these characteristics with the advantages of polyvinylidene fluoride and CS, Al-Gharabli et al. [59] achieved better separation performance of hybrid materials by adopting the method of “grafting”. The silicon alkyl modifier was used to fix CS on the surface of the porous structure and internally. Hydrophilic CS can improve the permeability of the membrane, imbue antifouling properties, and broaden the application range of the new materials. This is the first example of a chemical combination of CS with active polyvinylidene fluoride materials (Figure 10). 

## 5. Imitation Honeycomb Structure Material

**Figure 11:**
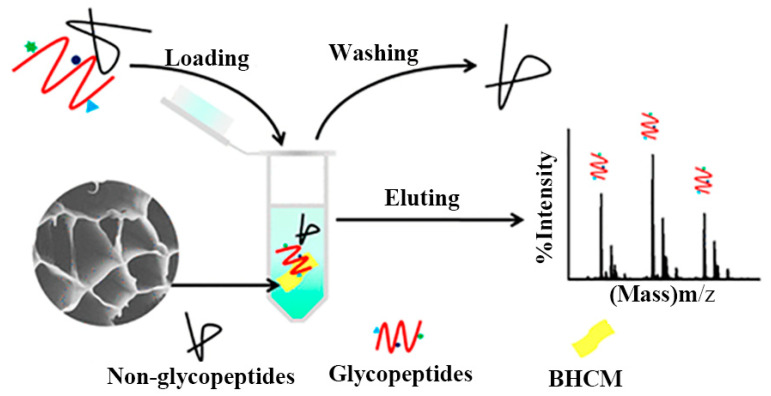
Schematic diagram of honeycomb-shaped macroporous bionic honeycomb chitosan film preparation with a freezing casting method. Reproduced with permission from [60], Copyrighter ACS publications 2019.

Based on indepth research into biomimetic material preparation and application, the honeycomb structure with the most tightly filled hexagonal holes in nature inspired to manufacture controllable micro-structure materials. Yu et al. [114] developed massively manufactured bionic polymer wood with a controllable honeycomb microstructure by combining traditional resin self-assembly and thermal curing. On this basis, Zhang et al. [60] prepared a new ceramic/polymer composite with honeycomb structure using CS and Poly(ethylene glycol) diglycidyl ether as precursors with a freezing casting method. The honeycomb structure effectively reduced steric hindrance between the material and glycopeptide (Figure 11). Three-dimensional honeycomb porous carbon has excellent mechanical properties and a particular surface area. Dai et al. exploited this property to prepare three-dimensional CS/honeycomb porous carbon/hydroxyapatite composite material. The synthesized composite scaffold had high porosity and specific mechanical strength similar to bone tissue and could promote osteogenesis [25]. To further expand the application potential of CS, Guo et al. [69] used it as the only combined source of carbon and nitrogen to controllably prepare an inherent nitrogen-doped honeycomb-like carbon/Fe_3_O_4_ composite material with favorable versatility. Notably, CS can spontaneously form a porous honeycomb-like structure after gelation and carbonization, so the composite material exhibited good microwave absorption properties. It also showed good ability to adsorb toxic metal ions Cr(VI) and degrade water organic pollutants. Deng et al. reported nitrogen-containing CS-based porous carbon microspheres (CPCM) with a honeycomb-like porous structure and a unique spherical morphology. The feasible step-by-step strategy proposed by the group under the conditions of HCl and KOH enabled the material’s structure to be precisely adjusted. It was also a promising reusable adsorbent with a high regenerative capacity [70]. Zhu et al. [71] prepared aminomethylphosphonic acid (AMPA) chelated cross-linked CS (CTSM@AMPA-Ti^4+^) composites with stable structure, low steric hindrance, and high Ti^4+^ loading. The material showed excellent selectivity and sensitivity to phosphopeptides and dimensional hindrance, which could be used as a promising adsorbent for the enrichment of phosphopeptides. To alleviate the increasingly severe environmental problems, Zou et al. [72] reported a novel eco-friendly material with high thermal conductivity and a honeycomb-like structure, which was composited by using the significant difference in scales between the CS microspheres (CSM) and the hydroxyl-functionalized hexagonal boron nitide (OH-BN) nanosheets. The novel heat-conduction materials were degradable, quick to recycle, and had a broad market prospect.

## 6. Conclusions

In this paper, the chitosan-based biomimetic materials based on the biological characteristics of bivalves, cell matrices, desert beetles, and honeycomb structure of bees were reviewed. Various forms of biomimetic functional materials (scaffolds, gels, films, and others) based on chitosan have promising applications in the fields of biomedical and adsorptive materials, but many challenges remain in this area. On the one hand, the functional mechanism of some organisms has not yet been fully understood. For example, the biomineralization mechanism of mollusks, such as shellfish, is not yet clear, and bionics is still in its preliminary stages. On the other hand, the long-term biosafety of the prepared materials is not yet clear, for example, whether the retention of the prepared materials loaded with growth factors or drugs will pose a risk to normal tissue growth. Based on the good biological properties and easy modification of chitosan, we believe that a better understanding of the functional mechanisms and safety of the materials involved in the above mentioned organisms, as well as the development of bionics, will greatly benefit the design of intelligent and biosecurity chitosan-based biomimetic materials. We expect that this review will be truly beneficial to the work of aspiring researchers in the field of CS bionics and its related applications.

## Figures and Tables

**Table 1:** Proposed applications of chitosan-based materials based on forms.

Forms	Composites	Applications	Ref.
Scaffold	CS, porous poly(ε-caprolactone) (PCL), bioactive glass (BG) polydopamine (PDA)	bone tissue engineering	[21]
	CS, graphene oxide (GO)	bone tissue engineering	[22]
	CS, poly (l-lactic acid) aligned microfibrous bundle	bioengineering	[23]
	CS, poly (methyl methacrylate-co-methacrylic acid) (P[MMA-co-MAA]), carbodiimide-crosslinker	bone tissue engineering	[24]
	CS, honeycomb porous carbon (HPC), nano-sized hydroxyapatite (nHA),	bioengineering	[25]
Hydrogel	CS, catechol	biomedical fields	[26]
	CS, CS-methacrylate (CS-MA), dopamine (DA), N-methylol acrylamide (NMA)	wound healing	[27]
	CS, HBC, DOPA	wound dressing	[28]
	CS, DA chloride	wound healing	[29]
	CS, catechol	biomedical fields	[30]
	CS, catechol	biomedical fields	[31]
	tetra-succinimidyl carbonate polyethylene glycol (PEG-4S), thiol-grafted mussel inspired catechol conjugated chitosan (CSDS)	wound healing	[32]
	CS, methacrylate modified CS, gelatin	wound healing	[33]
	CS-c, thiolated pluronic F-127	tissue engineering	[34]
	3, 4-dihydroxyhydrocinnamic acid glycol chitosan(g-CS), CS catechol (CS-c)	biomedical fields	[35]
	CS, catechol, diatom	biomedical fields	[36]
	chitosan quaternary ammonium salt (HTCC), oxidized dextran-dopamine (OD-DA)	wound healing	[37]
	glycol chitosan (GC), ciprofloxacin (Cip), PDA nanoparticles (NPs)	wound healing	[38]
	hydroxybutyl chitosan (HBC),l-dopamine (l-DOPA), ε-poly-l-lysine (EPL)	wound healing	[39]
	CS-C, β glycerol phosphate (β-GP), oyster peptides (OP)	wound dressing	[40]
	hydrocaffeic acid (HCA)-CS, iron oxide (γ-Fe_2_O_3_) MNPs	biomedical fields	[41]
	CS, oxidized hyaluronic acid (HAox) catechol terpolymer, Fe	wound dressing	[42]
	CS, collagen	biomedical fields	[43]
	CS, gelatin, compounded calcium phosphate (CCP)	bone tissue engineering	[44]
	combiningcarboxymethyl chitosan (CMCh), amorphous calcium phosphate (ACP)	bone tissue engineering	[45]
Film	CS, layered double hydroxides (LDHs),	materials design	[46]
	CS, CaCO_3_, Al_2_O_3_ alumina platelets	hybrid materials	[47]
	CS, PDA, silk fibroin nanofibers (SF)	biomedical fields	[48]
	CS, delignificated nano-cellulose (DNLC), MoS_2_	materials	[49]
	CS, HCA, BGNP, catechol	biomedical fields	[50]
	CS, alumina sheets	bionanocomposite	[51]
	CS, montmorillonite (MTM)	bionanocomposite	[52]
	O-carboxymethyl CS (CCS), MMT	fireproof materials	[53]
	CS, MMT	fireproof materials	[54]
	CS, poly (vinyl acetate) (PVAc), tetracycline (TC)	biomedical fields	[55]
	CCS, 2-methylacrylloxyethyl phosphorycholine (MPC), PDA, GRGDY peptide	biomedical fields	[56]
	CS, MTM, metal ions	bionanocomposite	[57]
	CS-gelatin (C:G), anodic alumina molds (AAM)	bone tissue engineering	[58]
	CS, poly (vinylidene fluoride) (PVDF)	wastewater treatment	[59]
	CS, poly (ethylene glycol) diglycidyl ether (PEGDGE, Mn = 500)	glycoproteomics	[60]
Others	CS-c, iron oxide nanoparticles (IONPs)	biomedical fields	[61]
	carbon nanotubes (CNT), carboxymethyl CS, PDA	efficient adsorbents	[62]
	CS, Fe_3_O_4_, PDA	efficient adsorbents	[63]
	CMC, Fe_3_O_4_, PDA	efficient adsorbents	[64]
	CS, CaCl_2_, Na_2_HPO_4_,	bone tissue engineering	[65]
	CS, hydroxyapatite (HA),	heavy metal removal	[66]
	CS, cis-butenediolic acid (maleic acid, MAc)	biomedical applications	[67]
	CS, fragments of human collagen I	biomedical fields	[68]
	CS, iron chloride hexahydrate (FeCl_3_·6H_2_O)	microwave absorbing	[69]
	CS, HCL, KOH	absorbing materials	[70]
	CS, aminomethyl phosphate, Ti^4+^	absorbing materials	[71]
	CS, hydroxyl-functionalized hexagonal boron nitide (OH-BN)	environment-friendly materials	[72]

## Data Availability

Not applicable.
